# Differential Expression of Sphingolipid Metabolizing Enzymes in Spontaneously Hypertensive Rats: A Possible Substrate for Susceptibility to Brain and Kidney Damage

**DOI:** 10.3390/ijms22073796

**Published:** 2021-04-06

**Authors:** Giuseppe Pepe, Maria Cotugno, Federico Marracino, Susy Giova, Luca Capocci, Maurizio Forte, Rosita Stanzione, Franca Bianchi, Simona Marchitti, Alba Di Pardo, Sebastiano Sciarretta, Speranza Rubattu, Vittorio Maglione

**Affiliations:** 1 IRCCS Neuromed, Pozzilli 86077, Italy; g.pepe1604@gmail.com (G.P.); maria.cotugno@neuromed.it (M.C.); federicomarracino@hotmail.it (F.M.); susygiova95@gmail.com (S.G.); luca.capocci@virgilio.it (L.C.); maurizio.forte@neuromed.it (M.F.); stanzione@neuromed.it (R.S.); franca.bianchi@neuromed.it (F.B.); simona.marchitti@neuromed.it (S.M.); 2 Department of Medical-Surgical Sciences and Biotechnologies, Sapienza University of Rome, Latina 04100, Italy; sebastiano.sciarretta@uniroma1.it; 3 Department of Clinical and Molecular Medicine, School of Medicine and Psychology, Sapienza University, Rome 00185, Italy

**Keywords:** sphingolipids, stroke, brain, kidney, SHR, WKY

## Abstract

Alterations in the metabolism of sphingolipids, a class of biologically active molecules in cell membranes with direct effect on vascular homeostasis, are increasingly recognized as important determinant in different vascular disorders. However, it is not clear whether sphingolipids are implicated in the pathogenesis of hypertension-related cerebrovascular and renal damage. In this study, we evaluated the existence of possible abnormalities related to the sphingolipid metabolism in the brain and kidneys of two well validated spontaneously hypertensive rat strains, the stroke-prone (SHRSP) and the stroke-resistant (SHRSR) models, as compared to the normotensive Wistar Kyoto (WKY) rat strain. Our results showed a global alteration in the metabolism of sphingolipids in both cerebral and renal tissues of both hypertensive strains as compared to the normotensive rat. However, few defects, such as reduced expression of enzymes involved in the metabolism/catabolism of sphingosine-1-phosphate and in the de novo biosynthetic pathways, were exclusively detected in the SHRSP. Although further studies are necessary to fully understand the significance of these findings, they suggest that defects in specific lipid molecules and/or their related metabolic pathways may likely contribute to the pathogenesis of hypertensive target organ damage and may eventually serve as future therapeutic targets to reduce the vascular consequences of hypertension.

## 1. Introduction

Hypertension represents a major health issue that causes specific organ damage and dramatic clinical consequences, leading to an increased rate of death and disability in Western societies [1,2,3]. To reduce the burden of the hypertensive complications, a more comprehensive understanding of the underlying etiopathogenetic mechanisms is required.

The availability of animal models that may closely mimic the human pathology and, at the same time, simplify the complexity inherent in the study of human populations, may be extremely useful in this contest. Currently, the stroke-prone spontaneously hypertensive rat (SHRSP), which displays a major predisposition to cerebral and renal damage, particularly when fed with a high-salt/low-potassium diet, represents an ideal model. Hypertension and dietary factors behave as predisposing risk factors for both rats and humans [4,5]. The SHRSP shows a higher frequency of stroke when fed with a Japanese style diet (JD) than its strictly related control, the stroke-resistant (SHRSR), despite similar blood pressure levels [6]. Of note, renal damage always precedes stroke events [6]. Although the underlying molecular mechanisms are not completely understood, evidence indicates that the resulting vascular dysfunction and the consequent structural lesions favor renal damage and stroke development. 

Over the last years, some of the mechanistic aspects and determinants of cerebrovascular and renal damage occurrence has been identified in the SHRSP model [7,8,9,10,11,12]. However, much still remains to be discovered. 

Sphingolipids, including Ceramides (Cer), sphingosine and its phosphorylated form Sphingosine-1-phosphate (S1P), represent the major lipid constituents of biological membranes and modulate several important functions for neuronal and non-neuronal cell populations [13]. No less important, however, is the pivotal role they play in the vascular formation and function [13,14,15], as well as in the regulation of blood brain barrier (BBB) integrity through the modulation of tight junction proteins [16]. In this regard, evidence indicates that de novo synthesis of sphingolipids is crucial in preserving endothelial cell functions [17,18], likely contributing to vascular integrity and homeostasis. 

Defective sphingolipid metabolism has been described to underlie different cardiovascular conditions [19,20]. However, no direct evidence of a causative role of sphingolipids in the pathogenesis of hypertensive target organ damage has been provided yet. 

Despite the lack of mechanistic details, the pathway of S1P, a bioactive lipid, has been shown to be neuroprotective during ischemia [21,22], and stimulation of its receptors leads to beneficial effects in an animal model of stroke [23]. Thus, it is conceivable that a tight interaction between stroke and sphingolipids exists. 

In this study, we investigated the cerebral and renal expression of several molecules involved in the sphingolipid pathways in both SHRSP and SHRSR strains, with respect to the normotensive WKY strain.

Our findings highlight, for the first time, an aberrant metabolism of sphingolipids either in the brain or in the kidney of both hypertensive animal models. Importantly, some defects were exclusively detected in the SHSRP strain. This evidence suggests that alterations in specific lipid molecules and/or their specific pathways may potentially contribute to the pathogenesis of hypertension and of hypertensive target organ damage. 

## 2. Results

### 2.1. Levels of S1P-Metabolizing Enzymes Are Perturbed in SHRSP

**Figure 1:**
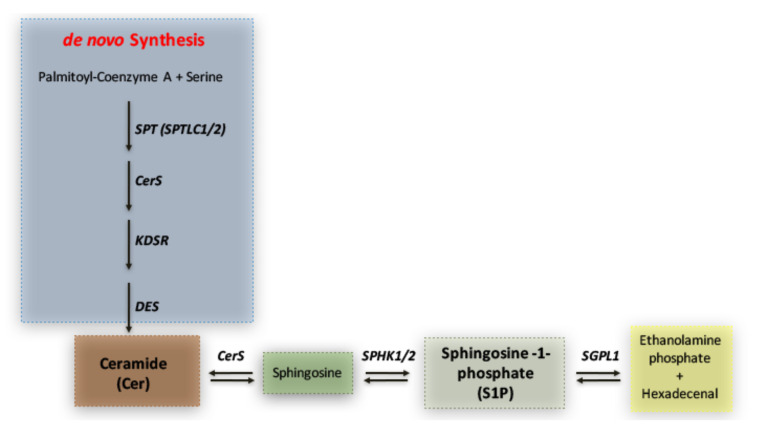
Simplified schematic representation of sphingolipid metabolism. Ceramide (Cer) represents the hub in the synthesis of sphingolipids. It is generated through the de novo biosynthetic route by the action of multiple enzymes such as Serine palmitoyltransferase (SPT) 3-keto-dihydrosphingosine reductase (KDSR), ceramide synthase (CERS) and ceramide desaturase (DES). Ceramide may also derive from sphingosine, which, in turn, produces sphingosine-1-phosphate (S1P) through phosphorylation by SPHK1/2. S1P can be irreversibly catabolized into hexadecenal + phospho-ethanolamine by S1P-Lyase (SGPL1).

To reveal whether any potential disturbance in the sphingolipid metabolism was detectable in SHRSP and SHRSR strains, we first investigated the expression of molecules implicated in S1P synthesis and degradation pathways (schematized in Figure 1). 

To this end, we assessed levels of SPHK1 and 2 as well as of SGPL1 in cerebral and renal tissues from both spontaneously hypertensive rats as compared to the WKY strain.

**Figure 2:**
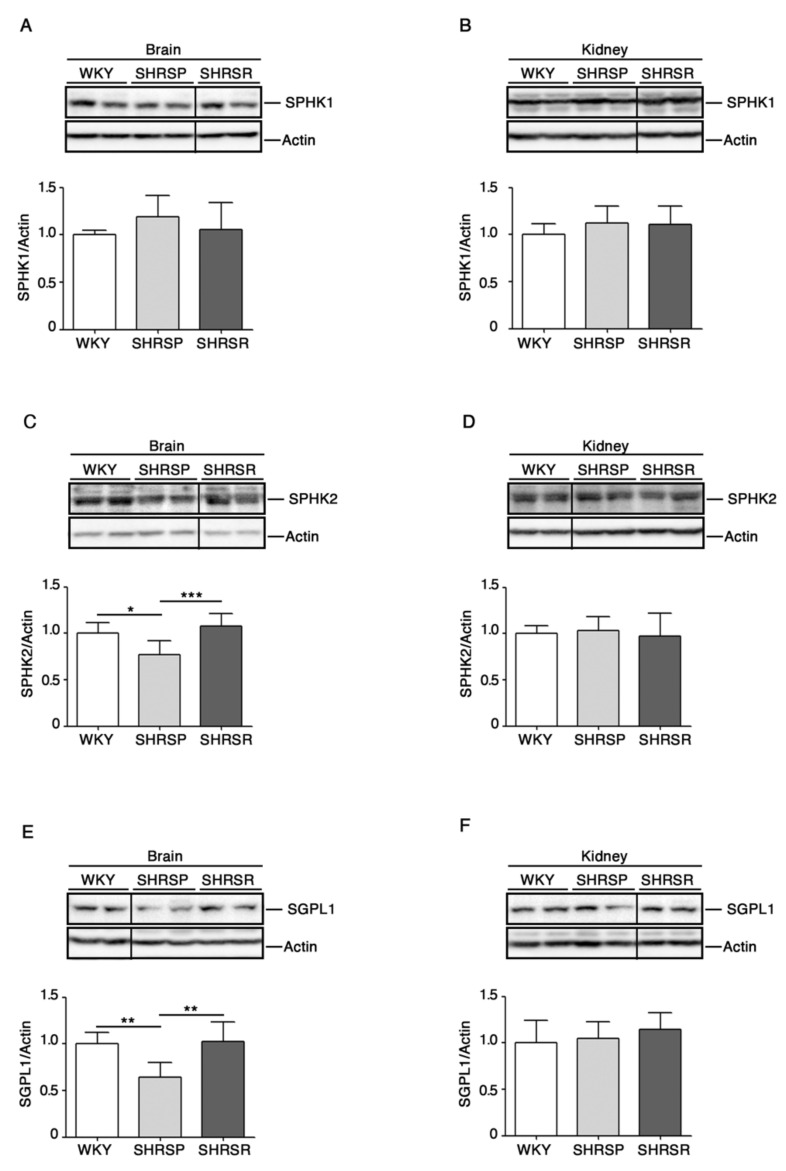
Expression of S1P metabolizing enzymes is defective in brain tissues from SHRSP. Representative cropped immunoblottings and densitometric analysis of SPHK1 (sphingosine kinase 1) (**A**,**B**), SPHK2 (sphingosine kinase 2) (**C**,**D**) and SGPL1 (S1P-Lyase) (**E**,**F**) in brain and kidney tissues from WKY, SHRSP and SHRSR. In each immunoblotting, all samples were run on the same gel. Non-adjacent samples are separated by a black line. Data are represented as mean ± SD, *n* = 6/7 for each group of rats. * *p* < 0.05; ** *p* < 0.01; *** *p* < 0.001 (one-way ANOVA, Tukey post-test). The raw date for generating the figure are reported in the Supplementary Material Table S1.

As reported in Figure 2, SHRSP rats were characterized by a significant reduction of SPHK2 as well as of SGPL1 in the brain when compared to both WKY and SHRSR. No difference in the renal expression of all enzymes was detected among the three rat strains.

### 2.2. Levels of S1P Receptors Are Aberrant in Spontaneously Hypertensive Rats

Evidence indicates that modulation of S1P receptors may be beneficial in ischemic models [23].

Here, in order to establish whether any change in their expression could be eventually associated with the disease phenotypes, we assessed protein levels of the most abundant receptors, S1PR1-3 [24], in the three rat strains.

**Figure 3:**
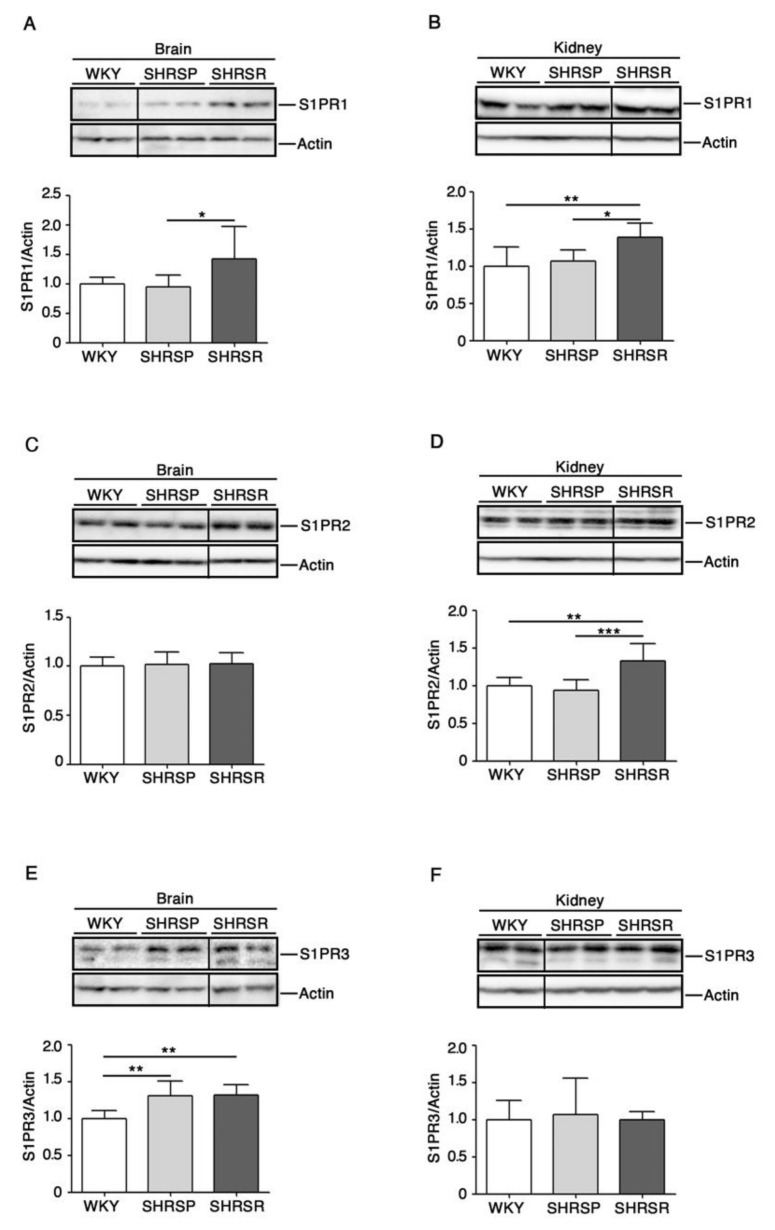
Expression of S1P receptors is aberrant in both brain and kidney tissues from SHRSP and SHRSR. Representative immunoblottings and densitometric analysis of S1PR1 (S1P receptor 1) (**A**,**B**), S1PR2 (S1P receptor 2) (**C**,**D**) and S1PR3 (S1P receptor 3) (**E**,**F**) in brain and kidney tissues from WKY, SHRSP and SHRSR. In each immunoblotting, all samples were run on the same gel. Non-adjacent samples are separated by a black line. Data are represented as mean ± SD, *n* = 6/8 for each group of rats. * *p* < 0.05; ** *p* < 0.01; *** *p* < 0.001 (one-way ANOVA, Tukey post-test). The raw date used for generating the figure are reported in the Supplementary Materials Table S2.

Immunoblotting analysis revealed a variation in the expression of all three receptors in a tissue-dependent manner. Interestingly, S1PR1 was the only receptor whose expression increased in both brain and kidneys of SHRSR (Figure 3A,3B). Conversely, the analysis of SHRSP tissues showed no variation in the expression of all receptors, except for S1PR3 whose levels increased in the brain, similarly to those detected in the SHRSR (Figure 3E).

### 2.3. Expression of Ceramide Synthases Is Defective in Multiple Tissues from SHRSP and SHRSR Models

Previous evidence indicates that levels of ceramide are increased in pre-clinical models of stroke as well as in human patients [25,26,27]. 

Here, in order to assess whether ceramide metabolism might be affected in spontaneously hypertensive rats, eventually resulting in increased levels of ceramide, we investigated the expression of the ceramide synthases, which are reported to be the most abundant in the brain (CerS1 and CerS2) and in the kidneys (CerS2 and CerS4) [28].

**Figure 4:**
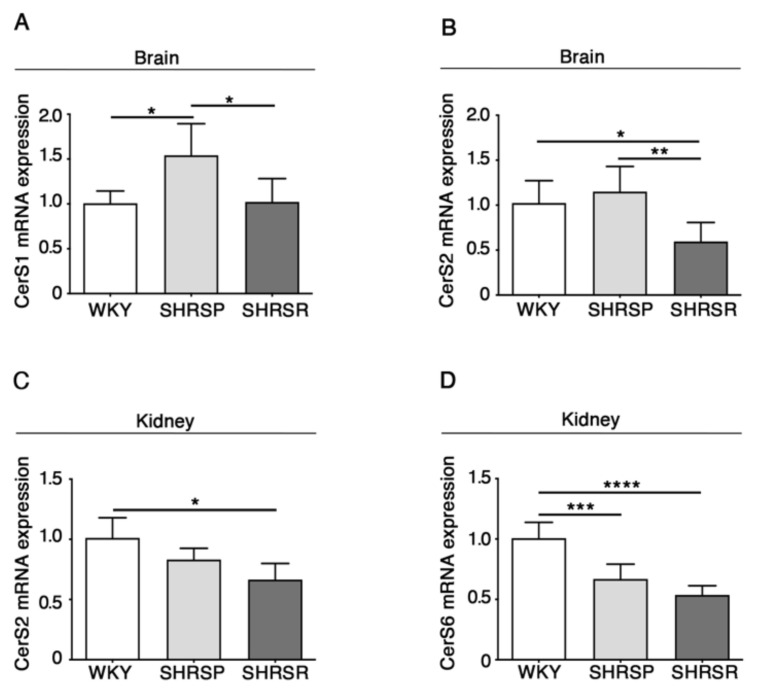
Gene expression of ceramide synthetizing enzymes is defective in brain and kidney tissues from SHRSP and SHRSR. qPCR analysis of CerS1 (ceramide synthase 1) (**A**) and CerS2 (ceramide synthase 2) (**B**) in brain tissue from WKY, SHRSP and SHRSR. qPCR analysis of CerS2 (**C**) and CerS6 (ceramide synthase 6) (**D**) in kidney tissue from WKY, SHRSP and SHRSR. Data are represented as mean ± SD, *n* = 5/6 for each group of rats. * *p* < 0.05; ** *p* < 0.01; *** *p* < 0.001; **** *p* < 0.0001 (one-way ANOVA, Tukey post-test). The raw date used for generating the figure are reported in the Supplementary Material Table S3.

As reported in Figure 4, qPCR analysis revealed a generalized derangement in the expression of the synthases, with some differences between the two spontaneously hypertensive strains. SHRSP showed increased levels of CerS1 mRNA in the brain when compared to either WKY or SHRSR (Figure 4A) and reduced levels of CerS6 in the kidneys, similarly to that observed in the SHRSR (Figure 4D). Conversely, SHRSR were characterized by a reduced expression of CerS2 mRNA in both brain and kidneys (Figure 4B,4C).

### 2.4. Expression of SPTLC1 Is Reduced in SHRSP Tissues

Recent studies have pointed out a potential involvement of de novo biosynthesis of sphingolipids in hypertension and vascular diseases [29,30].

To investigate any possible association between the de novo sphingolipid pathways and stroke, we assessed protein expression profiles of SPTLC1 and 2, two subunits of SPT, the rate-limiting enzymes in the de novo biosynthesis of sphingolipids [13], in both cerebral and renal tissues.

**Figure 5:**
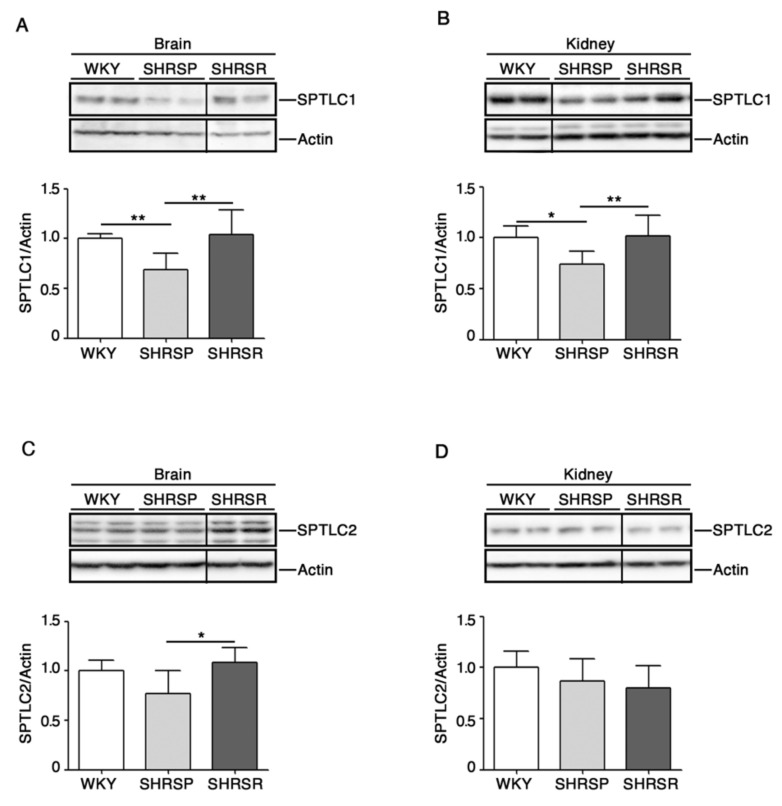
Expression of rate-limiting enzyme of de novo sphingolipid biosynthesis is defective in brain and kidney tissues from SHRSP and SHRSR. Representative immunoblottings and densitometric analysis of SPTLC1 (serine palmitoyltransferase long chain base subunit 1) (**A**,**B**) and SPTLC2 (serine palmitoyltransferase long chain base subunit 2) (**C**,**D**) in brain and kidney tissues from WKY, SHRSP and SHRSR. In each immunoblotting, all samples were run on the same gel. Non-adjacent samples are separated by a black line. Data are represented as mean ± SD, *n* = 6/7 for each group of rats. * *p* < 0.05; ** *p* < 0.01 (one-way ANOVA, Tukey post-test). The raw date used for generating the figure are reported in the Supplementary Materials Table S4.

As reported in Figure 5A,B, SHRSP rats were characterized by a significant reduction of SPTLC1 in both brain and kidneys. Interestingly, SPTCL2 was also reduced in the brain of SHRSP as compared to SHRSR (Figure 5C).

### 2.5. Expression of 3-Keto-Dihydrosphingosine Reductase Is Reduced in Both Spontaneously Hypertensive Rats

The alteration in the de novo biosynthesis of sphingolipids was further confirmed by the analysis of the enzymes 3-keto-dihydrosphingosine reductase (KDSR) and ceramide desaturase (DES), normally involved in the de novo synthesis of sphingolipids [13].

**Figure 6:**
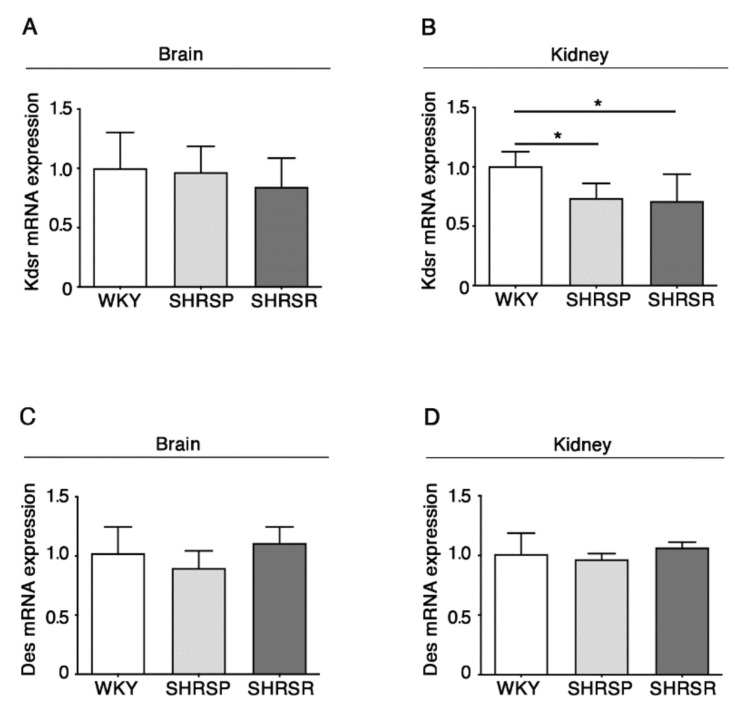
Expression of Kdsr mRNA is reduced in kidney tissues from SHRSP and SHRSR. qPCR analysis of Kdsr (3-keto-dihydrosphingosine reductase) (**A**,**B**) and Des (ceramide desaturase) (**C**,**D**) in brain and kidney tissues from WKY, SHRSP and SHRSR. Data are represented as mean ± SD, *n* = 5/6 for each group of rats. * *p* < 0.05 (one-way ANOVA, Tukey post-test). The raw date used for generating the figure are reported in the Supplementary Materials Table S5.

qPCR analysis showed a significant reduction of mRNA expression levels of KDSR in the kidneys of both SHRSP and SHRSR (Figure 6B). No further changes were observed.

## 3. Discussion

Despite tremendous research efforts by basic and clinical scientists, hypertensive complications such as stroke and renal diseases still represent major leading causes of death worldwide [1,2,3]. Thus, knowledge of alternative pathogenic pathways and new drug targets are needed to preserve cerebrovascular and kidney health. Exploring the metabolism of sphingolipids, a class of biologically active molecules in cell membranes that play a key role in vascular homeostasis, may pave the way towards new horizon of understanding. 

Although emerging evidence indicates that alterations in the metabolism of sphingolipids, like S1P and ceramides, may have a role in vascular disorders and hypertension [31], whether they are implicated in the pathogenesis of hypertensive organ damage still remains unclear. 

In this study, we attempted to gain some insights into the metabolism of sphingolipids in the SHRSP and SHRSR rat strains, two spontaneously hypertensive pre-clinical models with a different susceptibility to renal and cerebrovascular damage development. In particular, we performed the study in 8-week-old rats, at an age when they become hypertensive but are still free from target organ damage. Through this approach, we aimed to identify any “sphingobiological” signature potentially involved in the different outcome of the hypertensive disease in the two rat models. A normotensive rat strain served as the appropriate control.

Our findings showed for the first time that both hypertensive rat models were characterized by a global derangement of sphingolipid metabolism, but few changes were specific to either SHRSP or SHRSR strain.

In particular, the expression of both SPHK2 and SGPL1 was significantly reduced only in the brain of SHRSP. The reduction of SPHK2 levels may have extremely deleterious effects in the brain, where it is particularly abundant in the microvascular endothelial cells and exerts a protective role against the hypoxic preconditioning-induced ischemia [32]. On the other hand, the reduction of SGPL1 may be potentially toxic for neuronal cell functions [33].

In light of this, it is conceivable that an imbalance of S1P-metabolizing enzymes, with a consequent defective S1P bioavailability, may result in increased vascular damage. 

The possible involvement of S1P in the regulation of the molecular mechanisms potentially associated with stroke came from the evidence that levels of S1PR1, which has recently been identified as a positional candidate gene for salt-sensitivity in SHRSP [22], increased in the SHRSR tissues. 

These results suggest a possible double role of S1P in the determination of vascular damage and the consequent stroke event. In fact, the reduction of S1P bioavailability and/or the defective regulation of its related pathways could increase the susceptibility to stroke in the SHRSP. Conversely, increased expression of S1PR1, whose activation has been reported to be beneficial in experimental models of cerebral ischemia [34,35,36], could eventually represent a protective factor in SHRSR.

Our findings revealed another interesting difference between the two spontaneously hypertensive rat strains. The increased expression of CerS1, detected only in the SHRSP, may be suggestive of an elevated bioavailability of cerebral ceramide which may reveal toxic and potentially associated with increased susceptibility to stroke. This is corroborated by several studies reporting the association between increased ceramide content, small vessel ischemic disease and white matter hyper-intensities [37,38].

Another important observation derived from our study is the significant reduction of both SPTLC1 and 2 in SHRSP compared to SHRSR, in which no variations were detected. Although both hypertensive strains shared some defects in the expression of other genes involved in de novo sphingolipid synthesis, the reduction of SPTLCs clearly suggest a key role of this metabolic route in the pathogenesis of the cerebrovascular disease. In this context, recent evidence indicates that the endothelial de novo synthesis of sphingolipids regulates blood pressure and endothelial homeostasis [30], highlighting the critical role of de novo synthetized sphingolipids in vascular diseases. The current data may indicate that the de novo synthesis of sphingolipids could be perturbed in the SHRSP. 

Our study is the first addressing specific aspects of sphingolipid metabolism in regard to stroke. Although we did not perform any quantitative analysis of sphingolipids in rat tissues, our findings suggested a potential alteration in the bioavailability of different sphingolipids such as S1P and ceramide in SHRSP. Since S1P stimulates cell proliferation and survival pathways whereas ceramides usually exert anti-proliferative and apoptotic effects [39], the imbalance between them may affect brain homeostasis in SHRSP and potentially contribute to the pathogenesis of stroke. Considering the correlative nature of our study, further investigations are necessary to fully understand the potential pivotal role of sphingolipid alterations in the pathogenesis of hypertensive organ damage in order to develop more effective “sphingolipid-based” therapeutic strategies.

Considering that several drugs, whose molecular targets belong to sphingolipid pathways, are already available for the treatment of other diseases [40], we believe that our findings may offer support for exploring their therapeutic potential in hypertensive brain and renal vascular disorders.

## 4. Materials and Methods

### 4.1. Animal Models

All experiments were performed using two-month-old male stroke-prone spontaneously hypertensive (SHRSP), stroke-resistant spontaneously hypertensive (SHRSR) rat strains, and comparing them with an age- and sex-matched normotensive rat strain, Wistar-Kyoto (WKY). SHRSP and SHRSR are the most commonly used animal models in preclinical studies of hypertension and of associated stroke [5,6]. They were kept at the animal facility of the IRCCS Neuromed, maintained at constant room temperature and 12 h day/night cycles, and were fed with regular rat chow and water ad libitum. All animal studies were performed in accordance with approved protocols by the IRCCS Neuromed Animal Care Review Board and by “Istituto Superiore di Sanità” (ISS permit number: 1086/2020) and were conducted according to EU Directive 2010/63/EU for animal experiments.

### 4.2. Immunoblottings

Rats were sacrificed by decapitation and tissues (brain and kidneys) were snap-frozen in liquid N2 and pulverized in a mortar with a pestle. Pulverized tissue was homogenized in lysis buffer containing 20 mM Tris, pH 7,4, 1% Nonidet P-40, 1 mM EDTA, 20 mM NaF, 2 mM Na_3_VO_4_ and 1:1000 protease inhibitor mixture (Sigma-Aldrich) and sonicated with 2*10 s pulses. For immunoblotting, 20 µg of total protein lysate were immunoblotted with the following antibodies: anti-SPHK1 (1:1000) (Santa Cruz Biotechnology, Dallas, Texas, USA), anti-SPHK2 (1:1000) (Abcam, Cambridge, UK), anti-SGPL1 (1:1000) (Sigma-Aldrich, St. Louis, Missouri, USA), anti-S1PR1 (1:1000) (Immunological Sciences), anti-S1PR2 (1:1000) (Santa Cruz Biotechnology), anti-S1PR3 (1:1000) (Immunological Sciences), anti-SPTLC1 (1:1000) (Abcam) and anti-SPTLC2 (1:1000) (Abcam). For protein normalization, anti-actin (1:2000) (Immnunological Sciences, Rome, Italy) was used. Protein bands were detected by ECL Prime (GE Healthcare) and quantitated with Quantity One Software (Bio-Rad Laboratories, Hercules, CA, USA).

### 4.3. RNA Extraction and qPCR.

Total RNA was extracted using Rneasy kit (Qiagen) according to the manufacturer’s instructions. 1000 ng of total RNA was synthesized using Super Script III reverse transcriptase (Invitrogen) and the resulting cDNA was amplified using Power SYBR Green PCR Master Mix (Bio-Rad) following the manufacturer’s instructions. Quantitative qPCR analysis was performed using specific primers:

Kdsr FW: 5′-TAAAGCAGGCACAGGAGAAG-3′;

Kdsr RV: 5′-GCCTGAGAGGACACAAACA -3′;

Des FW: 5′-AGAGGAGTTCGAATGGGTCTA-3′;

Des RV: 5′- GATAGCCAGAGTCATGGAATGG-3′;

CerS1 FW: 5′- CCTGGAAGCTTCTGTTCTACTT-3′;

CerS1 RV: 5′- CATGTACACGGTGGCATAGA-3′;

CerS2 FW: 5′- CATGGCTGTCACTGTGGATAA-3′;

CerS2 RV: 5′- GAGAAGCAGAGGAGAATGATGG-3′;

CerS6 FW: 5′- CAGGAGTGGACAAAGCAAGA-3′;

CerS6 RV: 5′- TGGTTTGGCTATGAATCTCTCG-3′;

Gapdh FW: 5′- AACGACCCCTTCATTGACCTC-3′;

Gapdh RV: 5′- CCTTGACTGTGCCGTTGAACT-3′.

### 4.4. Statistics

One-way ANOVA followed by a Tukey post-test was used. Data are expressed as mean ± SD.

## Data Availability

The research raw data used for generating the figures are available as Supplementary Materials.

## Supplementary Materials

The following are available online at https://www.mdpi.com/article/10.3390/ijms22073796/s1. Table S1: Research raw data used for generating Figure 2. Table S2: Research raw data used for generating Figure 3. Table S3: Research raw data used for generating Figure 4. Table S4: Research raw data used for generating Figure 5. Table S5: Research raw data used for generating Figure 6.

## References

[1] Mills K.T., Stefanescu A., He J. (2020). The global epidemiology of hypertension. Nat. Rev. Nephrol.

[2] Benjamin E.J., Muntner P., Alonso A., Bittencourt M.S., Callaway C.W., Carson A.P., Chamberlain A.M., Chang A.R., Cheng S., Das S.R. (2019). Heart Disease and Stroke Statistics-2019 Update: A Report From the American Heart Association. Circulation.

[3] Rubattu S., Giliberti R., Volpe M. (2000). Etiology and pathophysiology of stroke as a complex trait. Am. J. Hypertens.

[4] Ascherio A., Rimm E.B., Hernan M.A., Giovannucci E.L., Kawachi I., Stampfer M.J., Willett W.C. (1998). Intake of potassium, magnesium, calcium, and fiber and risk of stroke among US men. Circulation.

[5] Yamori Y., Horie R., Tanase H., Fujiwara K., Nara Y., Lovenberg W. (1984). Possible role of nutritional factors in the incidence of cerebral lesions in stroke-prone spontaneously hypertensive rats. Hypertension.

[6] Rubattu S., Volpe M., Kreutz R., Ganten U., Ganten D., Lindpaintner K. (1996). Chromosomal mapping of quantitative trait loci contributing to stroke in a rat model of complex human disease. Nat. Genet..

[7] Rubattu S., Stanzione R., Volpe M. (2016). Mitochondrial Dysfunction Contributes to Hypertensive Target Organ Damage: Lessons from an Animal Model of Human Disease. Oxid Med. Cell Longev.

[8] Rubattu S., Stanzione R., Gigante B., Volpe M. (2004). Role of genetic factors in the etiopathogenesis of cerebrovascular accidents: From an animal model to the human disease. Cell Mol Neurobiol.

[9] Busceti C.L., Cotugno M., Bianchi F., Forte M., Stanzione R., Marchitti S., Battaglia G., Nicoletti F., Fornai F., Rubattu S. (2020). Brain Overexpression of Uncoupling Protein-2 (UCP2) Delays Renal Damage and Stroke Occurrence in Stroke-Prone Spontaneously Hypertensive Rats. Int. J. Mol. Sci..

[10] Rubattu S., Lee-Kirsch M.A., DePaolis P., Giliberti R., Gigante B., Lombardi A., Volpe M., Lindpaintner K. (1999). Altered structure, regulation, and function of the gene encoding the atrial natriuretic peptide in the stroke-prone spontaneously hypertensive rat. Circ. Res..

[11] Rubattu S., Stanzione R., Bianchi F., Cotugno M., Forte M., Della Ragione F., Fioriniello S., D’Esposito M., Marchitti S., Madonna M. (2017). Reduced brain UCP2 expression mediated by microRNA-503 contributes to increased stroke susceptibility in the high-salt fed stroke-prone spontaneously hypertensive rat. Cell Death Dis..

[12] Rubattu S., Di Castro S., Schulz H., Geurts A.M., Cotugno M., Bianchi F., Maatz H., Hummel O., Falak S., Stanzione R. (2016). Ndufc2 Gene Inhibition Is Associated With Mitochondrial Dysfunction and Increased Stroke Susceptibility in an Animal Model of Complex Human Disease. J. Am. Heart Assoc..

[13] Hannun Y.A., Obeid L.M. (2018). Sphingolipids and their metabolism in physiology and disease. Nat. Rev. Mol. Cell Biol..

[14] Hla T., Venkataraman K., Michaud J. (2008). The vascular S1P gradient-cellular sources and biological significance. Biochim. Biophys. Acta.

[15] Xiong Y., Hla T. (2014). S1P control of endothelial integrity. Curr. Top. Microbiol. Immunol..

[16] Cogolludo A., Villamor E., Perez-Vizcaino F., Moreno L. (2019). Ceramide and Regulation of Vascular Tone. Int. J. Mol. Sci..

[17] Wilkerson B.A., Argraves K.M. (2014). The role of sphingosine-1-phosphate in endothelial barrier function. Biochim. Biophys. Acta.

[18] Cantalupo A., Zhang Y., Kothiya M., Galvani S., Obinata H., Bucci M., Giordano F.J., Jiang X.C., Hla T., Di Lorenzo A. (2015). Nogo-B regulates endothelial sphingolipid homeostasis to control vascular function and blood pressure. Nat. Med..

[19] Sun N., Keep R.F., Hua Y., Xi G. (2016). Critical Role of the Sphingolipid Pathway in Stroke: A Review of Current Utility and Potential Therapeutic Targets. Transl. Stroke Res..

[20] Hasegawa Y., Suzuki H., Altay O., Rolland W., Zhang J.H. (2013). Role of the sphingosine metabolism pathway on neurons against experimental cerebral ischemia in rats. Transl. Stroke Res..

[21] Brait V.H., Tarrason G., Gavalda A., Godessart N., Planas A.M. (2016). Selective Sphingosine 1-Phosphate Receptor 1 Agonist Is Protective Against Ischemia/Reperfusion in Mice. Stroke.

[22] Graham D., McBride M.W., Gaasenbeek M., Gilday K., Beattie E., Miller W.H., McClure J.D., Polke J.M., Montezano A., Touyz R.M. (2007). Candidate genes that determine response to salt in the stroke-prone spontaneously hypertensive rat: Congenic analysis. Hypertension.

[23] Kraft P., Gob E., Schuhmann M.K., Gobel K., Deppermann C., Thielmann I., Herrmann A.M., Lorenz K., Brede M., Stoll G. (2013). FTY720 ameliorates acute ischemic stroke in mice by reducing thrombo-inflammation but not by direct neuroprotection. Stroke.

[24] Chun J., Hla T., Lynch K.R., Spiegel S., Moolenaar W.H. (2010). International Union of Basic and Clinical Pharmacology. LXXVIII. Lysophospholipid receptor nomenclature. Pharmacol. Rev..

[25] Chao H.C., Lee T.H., Chiang C.S., Yang S.Y., Kuo C.H., Tang S.C. (2019). Sphingolipidomics Investigation of the Temporal Dynamics after Ischemic Brain Injury. J. Proteome Res..

[26] Lee T.H., Cheng C.N., Chao H.C., Lee C.H., Kuo C.H., Tang S.C., Jeng J.S. (2021). Plasma ceramides are associated with outcomes in acute ischemic stroke patients. J. Formos Med. Assoc..

[27] Testai F.D., Hillmann M., Amin-Hanjani S., Gorshkova I., Berdyshev E., Gorelick P.B., Dawson G. (2012). Changes in the cerebrospinal fluid ceramide profile after subarachnoid hemorrhage. Stroke.

[28] Mullen T.D., Hannun Y.A., Obeid L.M. (2012). Ceramide synthases at the centre of sphingolipid metabolism and biology. Biochem. J..

[29] Sasset L., Zhang Y., Dunn T.M., Di Lorenzo A. (2016). Sphingolipid De Novo Biosynthesis: A Rheostat of Cardiovascular Homeostasis. Trends Endocrinol. Metab..

[30] Cantalupo A., Sasset L., Gargiulo A., Rubinelli L., Del Gaudio I., Benvenuto D., Wadsack C., Jiang X.C., Bucci M.R., Di Lorenzo A. (2020). Endothelial Sphingolipid De Novo Synthesis Controls Blood Pressure by Regulating Signal Transduction and NO via Ceramide. Hypertension.

[31] Borodzicz S., Czarzasta K., Kuch M., Cudnoch-Jedrzejewska A. (2015). Sphingolipids in cardiovascular diseases and metabolic disorders. Lipids Health Dis..

[32] Vessey D.A., Li L., Jin Z.Q., Kelley M., Honbo N., Zhang J., Karliner J.S. (2011). A sphingosine kinase form 2 knockout sensitizes mouse myocardium to ischemia/reoxygenation injury and diminishes responsiveness to ischemic preconditioning. Oxid. Med. Cell Longev..

[33] Hagen N., Van Veldhoven P.P., Proia R.L., Park H., Merrill A.H., van Echten- Deckert G. (2009). Subcellular origin of sphingosine 1-phosphate is essential for its toxic effect in lyase-deficient neurons. J Biol Chem..

[34] Iwasawa E., Ishibashi S., Suzuki M., Li F., Ichijo M., Miki K., Yokota T. (2018). Sphingosine-1-Phosphate Receptor 1 Activation Enhances Leptomeningeal Collateral Development and Improves Outcome after Stroke in Mice. J. Stroke Cerebrovasc. Dis..

[35] Nitzsche A., Poittevin M., Benarab A., Bonnin P., Faraco G., Uchida H., Favre J., Garcia-Bonilla L., Garcia M.C.L., Leger P.L. (2021). Endothelial S1P1 Signaling Counteracts Infarct Expansion in Ischemic Stroke. Circ. Res..

[36] Li Y.J., Shi S.X., Liu Q., Shi F.D., Gonzales R.J. (2020). Targeted role for sphingosine-1-phosphate receptor 1 in cerebrovascular integrity and inflammation during acute ischemic stroke. Neurosci. Lett..

[37] Azizkhanian I., Sheth S.A., Iavarone A.T., Lee S., Kakarla V., Hinman J.D. (2019). Plasma Lipid Profiling Identifies Biomarkers of Cerebral Microvascular Disease. Front. Neurol..

[38] Mielke M.M., Syrjanen J.A., Bui H.H., Petersen R.C., Knopman D.S., Jack C.R., Graff-Radford J., Vemuri P. (2019). Elevated Plasma Ceramides Are Associated With Higher White Matter Hyperintensity Volume-Brief Report. Arterioscl. Throm. Vas..

[39] Czubowicz K., Jesko H., Wencel P., Lukiw W.J., Strosznajder R.P. (2019). The Role of Ceramide and Sphingosine-1-Phosphate in Alzheimer’s Disease and Other Neurodegenerative Disorders. Mol. Neurobiol..

[40] Gonzalez-Cabrera P.J., Brown S., Studer S.M., Rosen H. (2014). S1P signaling: New therapies and opportunities. F1000Prime Rep..

