# Supplementary material for: Differential Expression of Sphingolipid Metabolizing Enzymes in Spontaneously Hypertensive Rats: A Possible Substrate for Susceptibility to Brain and Kidney Damage
Source: Int J Mol Sci. 2021 Apr 6;22(7):3796. doi: 10.3390/ijms22073796 (PMC8038804; doi:10.3390/ijms22073796)
Supplement: Supplementary file 1 [file ijms-22-03796-s001.pdf]

Supplementary Materials

Table S1. Research raw data used for generating Figure 2

Fig. 2A			
Brain	WKY	SHRSP	SHRSR
SPHK1/Actin	0,9404566	1,599703	1,31174
	1,012362	1,137452	0,687535
	1,047181	1,03609	1,394125
	1,049265	1,283598	1,278426
	1,005049	0,967141	1,072914
	0,9456854	1,112394	0,873326
			0,755608

Fig. 2B			
Kidney	WKY	SHRSP	SHRSR
SPHK1/Actin	1,080932	1,406951	1,369044
	1,093998	1,285679	1,315465
	0,8250692	1,085446	0,887453
	1,051587	1,002166	0,998396
	1,07878	0,977307	1,061202
	1,02888	0,964258	1,014346
	0,8407536		

Fig. 2C			
Brain	WKY	SHRSP	SHRSR
SPHK2/Actin	0,8101965	0,643296	1,167477
	1,191391	0,660055	0,999382
	0,9984123	0,584177	1,200412
	0,9954528	0,76696	1,23875
	0,9640843	0,910754	0,906435
	1,049686	0,932047	1,095093
	0,9907768	0,907666	0,952241

Fig. 2D	WKY	SHRSP	SHRSR
Kidney	0,9560941	1,11866	0,71441
SPHK2/Actin	1,152764	0,921135	0,889108
	1,013845	0,991965	0,631717
	0,8772969	0,827123	1,27449
	0,9678484	0,94815	1,199867
	1,056076	1,177415	0,957411
	0,9760756	1,249721	1,147893

Fig. 2E			
Brain	WKY	SHRSP	SHRSR
SGPL1/Actin	0,9243483	0,388135	0,696111
	1,068057	0,478552	1,048796
	1,007594	0,752438	1,101234
	1,169532	0,732762	1,057485
	1,045154	0,638241	0,887821
	0,9954996	0,650277	1,382341
	0,7898142	0,851075	1,019141

Fig. 2F			
Kidney	WKY	SHRSP	SHRSR
SGPL1/Actin	1,252236	1,245379	1,20521
	0,6525998	1,278968	1,164063
	1,095165	1,162333	1,501205
	1,208875	0,838322	1,115
	0,7673701	0,994958	1,054229
	1,023755	0,93508	1,049997
		0,876816	0,946886

Table S2. Research raw data used for generating Figure 3

Fig. 3A			
Brain	WKY	SHRSP	SHRSR
S1PR1/Actin	1,037206	1,151377	1,995708
	1,186173	1,084609	2,177769
	0,921421	1,111785	1,282252
	0,8552	0,776294	1,229511
	1,056409	0,87505	1,079444
	0,911215	0,643283	0,781973
	1,058344		
	0,974032		

Fig. 3B			
Kidney	WKY	SHRSP	SHRSR
S1PR1/Actin	0,8121079	0,928123	1,538605
	1,254523	1,064127	1,680846
	0,933369	0,915357	1,261811
	0,6890233	1,274984	1,323123
	1,332768	1,162414	1,201363
	1,177974	1,216978	1,510164
	0,8002344	0,913192	1,200468

Fig. 3C			
Brain	WKY	SHRSP	SHRSR
S1PR2/Actin	0,9720842	0,990761	0,908389
	1,122318	0,895176	1,077852
	0,9055977	1,15655	0,846179
	0,918593	0,841749	1,115427
	0,9981151	1,000188	1,038811
	1,135551	1,01965	1,106329
	0,9477406	1,209775	1,103557

Fig. 3D			
Kidney	WKY	SHRSP	SHRSR
S1PR2/Actin	0,9832389	0,869594	1,236219
	1,072379	0,834955	1,434172
	0,9443825	0,813219	1,600734
	0,8080044	0,866409	1,526205
	1,143765	1,137629	1,414281
	1,075171	1,12956	1,112413
	0,9730599	0,931034	0,995067

Fig. 3E			
Brain	WKY	SHRSP	SHRSR
S1PR3/Actin	1,166148	1,161502	1,420357
	0,9003416	1,252774	1,422551
	0,9974293	1,296696	1,116424
	0,9360809	1,224871	1,30212
	0,9879002	1,076159	1,43727
	0,9056146	1,523401	1,393428
	1,106485	1,632333	1,140901

Fig. 3F			
Kidney	WKY	SHRSP	SHRSR
S1PR3/Actin	0,9760419	1,5664	0,935749
	1,321565	1,681744	1,212494
	0,7023931	1,477414	0,96066
	1,243754	0,533232	0,916005
	1,143428	0,585973	0,918348
	0,9632587	0,815736	1,01134
	0,6495588	0,810117	1,052952

Table S3 Research raw data used for generating Figure 4

Fig. 4 A			
Brain	WKY	SHRSP	SHRSR
CerS1 mRNA expression	1,058	1,999	0,859
	0,892	1,511	1,377
	1,06	1,359	0,786
	0,848	1,756	1,235
	1,18	1,09	0,755
			1,126

Fig. 4 B			
Brain	WKY	SHRSP	SHRSR
CerS2 mRNA expression	1,159	1,37	0,343
	1,429	1,454	0,66
	0,787	0,741	0,943
	0,768	1,114	0,552
	0,961	1,074	0,425
	1,04		0,655

Fig. 4 C			
Kidney	WKY	SHRSP	SHRSR
CerS2 mRNA expression	0,894	0,76	0,579
	1,007	0,878	0,81
	0,994	0,917	0,776
	1,117	0,913	0,672
	1,269	0,843	0,491
	0,788	0,679	

Fig. 4 D			
Kidney	WKY	SHRSP	SHRSR
CerS6 mRNA expression	0,881	0,703	0,594
	0,902	0,718	0,487
	1,055	0,743	0,516
	1,193	0,814	0,652
	0,9	0,503	0,534
	1,111	0,536	0,435

Table S4. Research raw data used for generating Figure 5

Fig. 5 A			
Brain	WKY	SHRSP	SHRSR
SPTLC1/Actin	1,000762	0,5913	0,74723
	0,977372	0,499868	0,88475
	1,021867	0,633237	1,467964
	1,014579	0,53329	1,024956
	0,98555	0,824854	0,986082
	1,071548	0,916006	1,263941
	0,928323	0,834172	0,898149

Fig. 5 B			
Kidney	WKY	SHRSP	SHRSR
SPTLC1/Actin	0,817981	0,92582	0,816306
	0,990848	0,667117	1,295899
	1,150198	0,728644	1,238768
	1,040973	0,886519	1,132571
	1,10635	0,763907	0,926638
	0,966253	0,611491	0,850861
	0,927398	0,57639	0,852729

Fig. 5 C			
Brain	WKY	SHRSP	SHRSR
SPTLC2/Actin	0,991127	0,620984	1,09859
	1,109686	0,453217	1,302147
	0,899186	0,601085	0,977586
	0,932252	0,948132	1,289812
	1,160061	0,955952	1,007702
	0,907688	1,026975	0,964268
			0,951798

Fig. 5 D			
Kidney	WKY	SHRSP	SHRSR
SPTLC2/Actin	1,140124	0,794648	0,616402
	0,933301	0,63171	0,659012
	0,926574	0,702714	0,551422
	0,788972	0,694774	0,937982
	0,993027	0,985693	1,012699
	1,28293	1,076916	1,028436
	0,935072	1,19468	

Table S5. Research raw data used for generating Figure 6

Fig. 6 A			
Brain	WKY	SHRSP	SHRSR
Kdsr mRNA expression	1,29266	1,302193	1,044805
	0,985701	1,013346	0,546235
	1,326025	0,768351	0,990467
	0,489037	0,781697	1,104862
	0,949476	0,978074	0,805529
	0,957102		0,575786

Fig. 6 B			
Kidney	WKY	SHRSP	SHRSR
Kdsr mRNA expression	1,028	0,837	1,023
	1,014	0,848	0,611
	0,853	0,754	0,682
	1,124	0,816	0,945
	1,145	0,605	0,44
	0,873	0,561	0,569

Fig. 6 C			
Brain	WKY	SHRSP	SHRSR
Degs mRNA expression	1,031	1,092	1,196
	1,155	0,983	0,874
	1,282	0,715	1,27
	0,655	0,88	1,121
	0,891	0,822	1,143
	1,122		1,053

Fig. 6 D			
Kidney	WKY	SHRSP	SHRSR
Degs mRNA expression	1,187	0,969	1
	0,842	0,912	1,069
	0,86	0,923	1,03
	0,969	0,985	1,09
	0,947	0,978	1,122
	1,266	1,043	1,09
